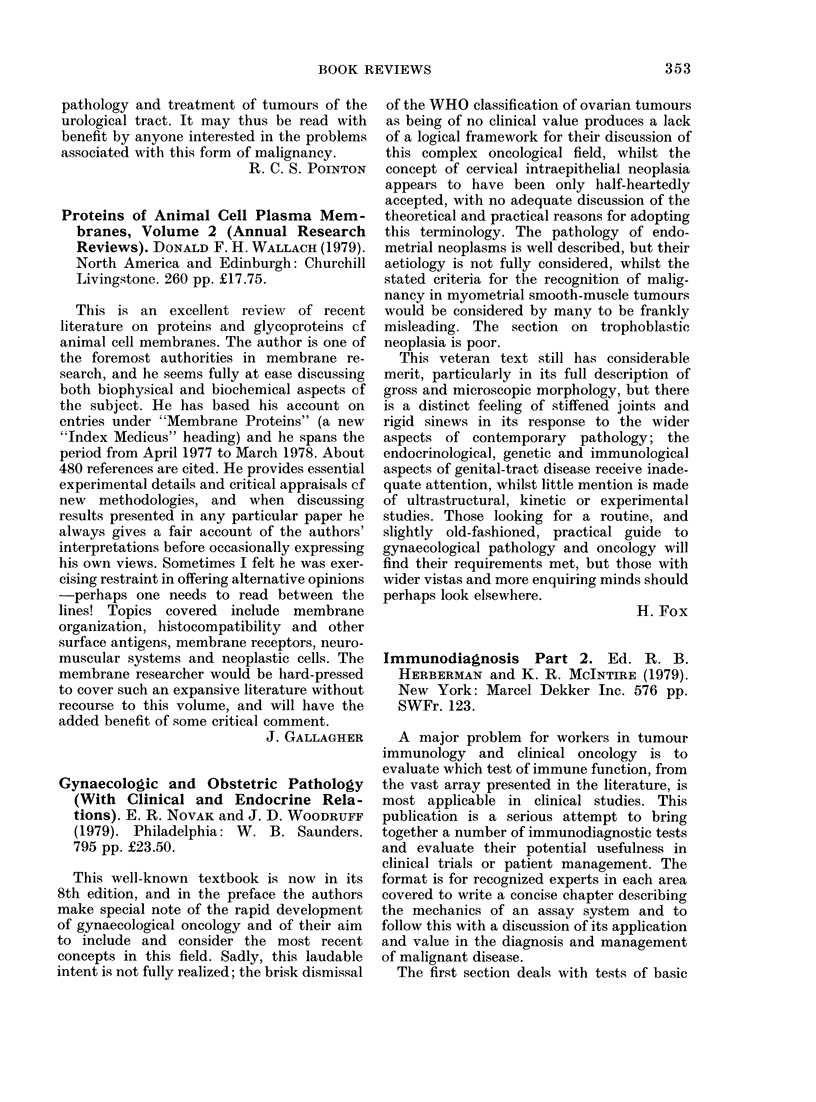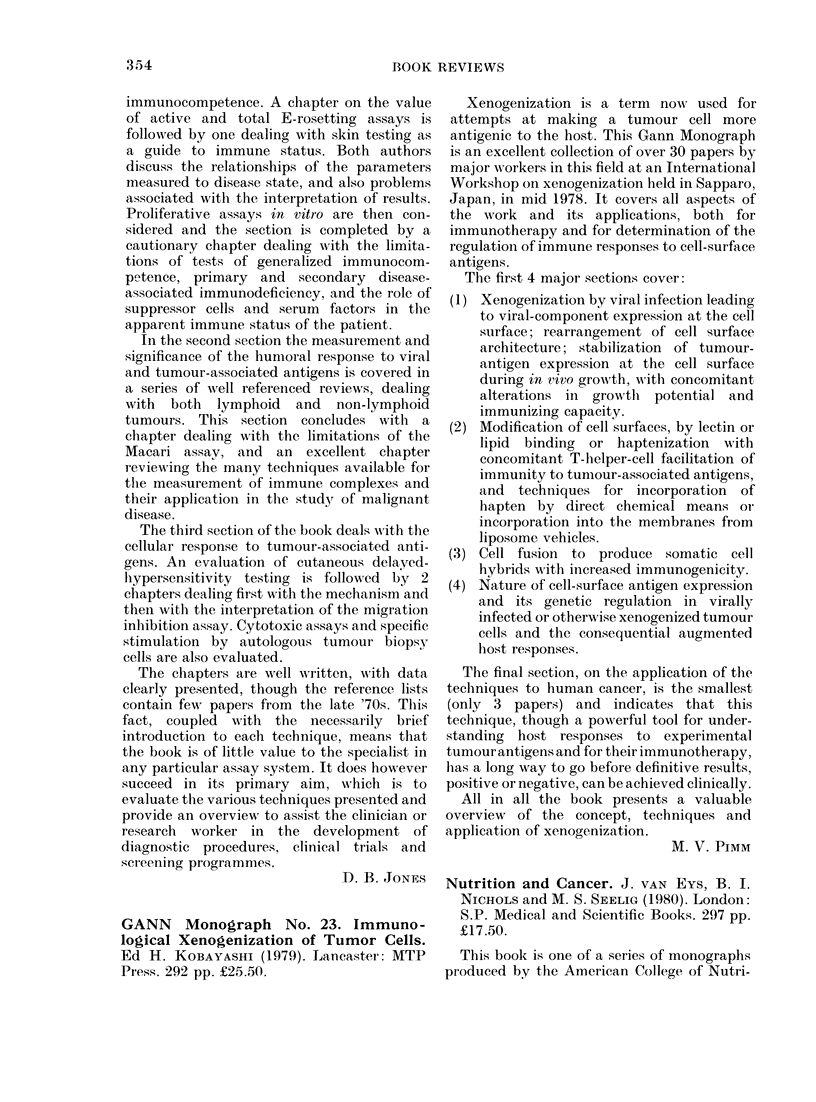# Immunodiagnosis Part 2

**Published:** 1980-08

**Authors:** D. B. Jones


**Immunodiagnosis Part 2.** Ed. R. B. HERBERMAN and K. R. McINTIRE (1979). New York: Marcel Dekker Inc. 576 pp. SWFr. 123.

A major problem for workers in tumour immunology and clinical oncology is to evaluate which test of immune function, from the vast array presented in the literature, is most applicable in clinical studies. This publication is a serious attempt to bring together a number of immunodiagnostic tests and evaluate their potential usefulness in clinical trials or patient management. The format is for recognized experts in each area covered to write a concise chapter describing the mechanics of an assay system and to follow this with a discussion of its application and value in the diagnosis and management of malignant disease.

The first section deals with tests of basic immunocompetence. A chapter on the value of active and total E-rosetting assays is followed by one dealing with skin testing as a guide to immune status. Both authors discuss the relationships of the parameters measured to disease state, and also problems associated with the interpretation of results. Proliferative assays *in vitro* are then considered and the section is completed by a cautionary chapter dealing with the limitations of tests of generalized immunocompetence, primary and secondary disease-associated immunodeficiency, and the role of suppressor cells and serum factors in the apparent immune status of the patient.

In the second section the measurement and significance of the humoral response to viral and tumour-associated antigens is covered in a series of well referenced reviews, dealing with both lymphoid and non-lymphoid tumours. This section concludes with a chapter dealing with the limitations of the Macari assay, and an excellent chapter reviewing the many techniques available for the measurement of immune complexes and their application in the study of malignant disease.

The third section of the book deals with the cellular response to tumour-associated antigens. An evaluation of cutaneous delayed-hypersensitivity testing is followed by 2 chapters dealing first with the mechanism and then with the interpretation of the migration inhibition assay. Cytotoxic assays and specific stimulation by autologous tumour biopsy cells are also evaluated.

The chapters are well written, with data clearly presented, though the reference lists contain few papers from the late '70s. This fact, coupled with the necessarily brief introduction to each technique, means that the book is of little value to the specialist in any particular assay system. It does however succeed in its primary aim, which is to evaluate the various techniques presented and provide an overview to assist the clinician or research worker in the development of diagnostic procedures, clinical trials and screening programmes.

D. B. JONES